# Compressive Sensing-Based Bandwidth Stitching for Multichannel Microwave Radars

**DOI:** 10.3390/s20030665

**Published:** 2020-01-24

**Authors:** Paul Berry, Ngoc Hung Nguyen, Hai-Tan Tran

**Affiliations:** 1 Intelligence, Surveillance and Space Division, Defence Science and Technology Group, Edinburgh, SA 5111, Australia; haitan.tran@dst.defence.gov.au; 2 Maritime Division, Defence Science and Technology Group, Edinburgh, SA 5111, Australia; ngoc.nguyen@dst.defence.gov.au

**Keywords:** radar signal processing techniques, radar imaging, multiband processing, compressive sensing, sparse reconstruction, bandwidth stitching

## Abstract

The problem of obtaining high range resolution (HRR) profiles for non-cooperative target recognition by coherently combining data from narrowband radars was investigated using sparse reconstruction techniques. If the radars concerned operate within different frequency bands, then this process increases the overall effective bandwidth and consequently enhances resolution. The case of unknown range offsets occurring between the radars’ range profiles due to incorrect temporal and spatial synchronisation between the radars was considered, and the use of both pruned orthogonal matching pursuit and refined $l_{1}$-norm regularisation solvers was explored to estimate the offsets between the radars’ channels so as to attain the necessary coherence for combining their data. The proposed techniques were demonstrated and compared using simulated radar data.

## 1. Introduction

The construction of high range resolution profiles (HRRP) of targets is a precursor to feature extraction for automatic target recognition (ATR), and normally requires the employment of a high-bandwidth waveform following detection by a lower resolution radar mode. Examples of recent papers in the non-cooperative target recognition (NCTR) literature focusing on feature extraction for ATR following HRRP construction are [1,2,3]. This paper considers the problem of HRRP construction, but using low resolution radars operating in different frequency bands for the purpose of combining their signals to achieve a higher resolution, and examines the problem of their data not being mutually coherent.

The ability to acquire high resolution range profiles of targets has improved over time as hardware capability has developed, with higher resolution being achieved by increasing the time-bandwidth product. In the early approaches, for narrowband radars with very limited instantaneous bandwidth, stepped-frequency waveforms were used with a single I,Q (that is, baseband quadrature) signal sample received after each frequency step. The set of samples is effectively used for the Fourier transform of the slant range profile, enabling the range response to be obtained simply by implementing an inverse Fourier transform (see, e.g., [4]). The greater the frequency range, the higher the range resolution, but the downside is that the burst of pulses can be so long that a scatterer may migrate between range cells, causing smearing of the range profile, and therefore requiring range compensation. An example of a recent paper involving the use of stepped-frequency waveforms is [5], and recent papers which have investigated the effects of target motion and aspect sensitivity are [6,7].

An advance on the stepped-frequency approach is to increase the time-bandwidth product using stretch processing, whereby a wideband LFM waveform is transmitted, and pulse-compression is achieved in hardware by mixing the received signal with an extended replica of the transmitted waveform. A point target at a particular range will manifest itself as a single frequency which is proportional to its range. The result, which is digitally sampled in time in order to facilitate the identification of the frequency components, is therefore a superposition of discrete frequencies, each corresponding to a point target at a different range. An application of the inverse Fourier transform again recovers the range profile (see, e.g., [4,8,9]).

A spectral analysis technique to improve range resolution was proposed in [10,11] based on autoregressive linear prediction. The main idea is to combine the mutually coherent signals received from multiple waveforms transmitted sequentially or concurrently, which have widely separate carrier frequencies or may even occupy entirely different frequency bands. Viewed in the spectral domain, the received signals from individual waveforms may be seen to occupy discrete wavebands which are separate or contiguous. If contiguous, then they can potentially be coherently combined to synthesise the signals that would have been received from a single wider bandwidth waveform in the manner presented in [12]. If separate, then presumably this coherent combination of signals would still be feasible, as would be the interpolation of the frequency response in the gaps between the bands under the a priori assumption that the signals are returned from discrete scatterers using spectral estimation techniques (see, e.g., [13]). Alternatively, the signals from different frequency bands can be jointly processed without explicitly filling the gaps between the bands. Since no new synthetic frequency band is actually constructed, this approach can be referred to as bandwidth stitching to distinguish it from bandwidth interpolation and extrapolation. The main challenge of this approach lies in the presence of phase errors in different frequency bands resulting from post-processed motion compensation which is often carried out separately for each frequency band. In this paper, we focus on the problem of bandwidth stitching for radar high-resolution range profiling and explore the use of sparse reconstruction to deal with the phase error problem.

It is convenient to formulate these ideas in the spectral domain, within which point scatterers appear as discrete sinusoids and which are amenable to analysis by spectral estimation techniques such as autoregression, as presented in [10,11]. Compressive sensing and sparse reconstruction, however, provide for the possibility of alternative signal representations, potentially allowing for greater flexibility and discriminating between signals of physical origin and receiver noise [13,14,15,16,17,18,19,20]. Instances of non-sinusoidal signals are waveforms in fast-time and signals returned from rotating objects when the angle of rotation is large. Compressive sensing and sparse reconstruction can also handle the situation corresponding to data being non-uniformly sampled in time or space, such as non-uniform PRF (pulse repetition frequency) waveforms and random sparse arrays.

Compressive sensing and sparse reconstruction were exploited in [17,19,21,22] to address the problem of gaps in the data both in slow-time and in frequency for inverse synthetic aperture radar (ISAR) imaging. However, these works assumed that the data were coherent across different sub-bands and that there were no model uncertainties. The work [23] took account of the possible lack of mutual coherence between the radars operating on the different sub-bands arising from incorrect timing synchronisation, or, equivalently, errors in antenna phase’s centre-relative locations. This is achieved by fitting an ultra-wideband all-pole signal model to the mutually-coherent sub-bands, which is then used for bandwidth interpolation and extrapolation prior to recovering the range profile by means of an inverse Fourier transform. This paper, however, proposes the use of compressive sensing and sparse reconstruction to deal with the non-coherence problem between different sub-bands.

To address the sub-band non-coherence problem, two different approaches were explored: (i) greedy pursuit and (ii) $l_{1}$-norm regularisation. In the first approach, pruned orthogonal matching pursuit (POMP) [24], which was originally developed for micro-Doppler parameter estimation, is adopted to deal with the dictionary mismatch which is due to the phase errors in each sub-band resulting from the motion-compensation post processing. The main idea is to parameterise the dictionary as a function of the phase errors and to construct multiple realisations of the dictionary. A selective learning process is then used to discard the dictionaries which correspond to incorrect values of phase errors. Since a straight application of the POMP algorithm to the problem under consideration would have been computationally expensive, we first applied the POMP algorithm pairwise to sub-bands in order to estimate the phase errors, and then utilised the conventional OMP algorithm to determine the range profile based on the estimated phase error values. In the second approach, an $l_{1}$-norm regularisation problem can be solved jointly both for the range profile vector and the phase errors [25]. The work [25] offers two solutions for joint synthetic aperture radar (SAR) imaging and phase error correction. The first solution is not applicable to the problem under consideration because a constant phase error in each sub-band is assumed. On the other hand, the second solution considers general arbitrary phase errors, and thus can be applied to our problem. Since the phase errors within each sub-band are a linear function of the range error coming from post-processing motion compensation, we also present refined variants of the second solution of [25] to take into account this underlying structure of the phase error.

The paper is organised as follows. Section 2 formulates the problem of bandwidth stitching for HRRP in the presence of phase errors. The POMP algorithm is applied in Section 3 to the bandwidth stitching problem under consideration. Section 4 presents $l_{1}$-norm regularisation solvers. Numerical performance comparisons are provided in Section 5 and conclusions are drawn in Section 6.

## 2. Problem Formulation

Consider a multistatic radar system consisting of *M* radar channels on different and distinct frequency sub-bands, approximately co-located, and illuminating a common target, such that their radar lines of sight (LoS) coincide but their range profiles are out of alignment. Each channel can individually produce a one dimensional range profile of the target, but with relatively coarse resolution. The bandwidth stitching problem can be briefly stated as follows: for the *M* generally non-coherent channels, the aim is to coherently combine, or “stitch,“ the channels together so that they can effectively produce a single range profile with resolution corresponding to the combined overall signal bandwidth.

Let $f_{m,n}$ ($n=1,\ldots ,N_{m}$) denote the $n^{\mathrm{th}}$ frequency bin of the $m^{\mathrm{th}}$ channel (m=1,…,M). Here, $N_{m}$ is the number of frequency bins in the $m^{\mathrm{th}}$ channel. We base the formulation on the point-scatterer model and assume that the target can be defined as consisting of *K* scattering centres at local line-of-sight coordinates $x_{k}$ (or local “down ranges”) and having complex-valued reflectivity coefficients $\alpha_{k}$, which are also assumed frequency-independent. The down-converted, pulse-compressed, motion-compensated signals received in each channel, in the frequency domain, can be written as

(1) $$S_{m}={\left[\ldots ,S_{m,n},\ldots \right]}_{n=1,\ldots ,N_{m}}^{T}\;,$$

where superscript *T* denotes the transpose operation, and

(2) $$S_{m,n}={\left|A\left(f_{m,n}\right)\right|}^{2}\exp\left\{-\frac{4\pi jf_{m,n}}{c}\Delta R_{m}\right\}\sum_{k=1}^{K}\alpha_{k}\exp\left\{-\frac{4\pi jf_{m,n}}{c}x_{k}\right\}.$$

Here, $A(f_{n,m})$ represents the transmit radar waveform, the squared amplitude resulting from pulse compression processing; constant *c* denotes the speed of light; and $\Delta R_{m}$ accounts for the range errors in the motion-compensation processing. Bandwidth stitching in this context amounts to estimating these phase errors as accurately as possible.

The signals $S_{m}$ in (1) can be rewritten in a more compact form as

(3) $$S_{m}=\Lambda_{m}F_{m}^{†}\alpha^{†},$$

where

(4)Λm=diag…,exp−4πjfm,ncΔRm,…n=1,…,Nm(5)Fm†=[…,Fm,k,…]k=1,…,K(6)Fm,k†=…,exp−4πjfm,ncxk,…n=1,…,NmT(7)α†=[…,αk,…]k=1,…,KT

Here, “diag” denotes a diagonal matrix; $\Lambda_{m}$ is referred to as the phase error matrix, of dimension $N_{m}\times N_{m}$; $F_{m}^{†}$ and $\alpha^{†}$ are respectively, dimensions $N_{m}\times K$ and K×1; $S_{m}$ is a column vector of dimension $N_{m}\times 1$; and the dagger symbol † refers to the *K* actual scatterers on the target.

To apply the sparse representation techniques of compressive sensing, we discretise the target’s local range coordinate *x* using a regularly-spaced range grid $\left\{x_{l}\right\}$ for $l=1,\ldots ,L_{x}$, with $L_{x}\gg K$, and construct the $N_{m}\times L_{x}$ dictionary matrices

(8) $$F_{m}={\left[\ldots ,F_{m,l},\ldots \right]}_{l=1,\ldots ,L_{x}}\;,$$

where

(9) $$F_{m,l}={\left[\ldots ,\exp\left\{-\frac{4\pi jf_{m,n}}{c}x_{l}\right\},\ldots \right]}_{n=1,\ldots ,N_{m}}^{T}$$

Are the “atoms” of dictionary $F_{m}$ in the frequency domain. The corresponding range profile vector

(10) $$\alpha ={\left[\ldots ,\alpha_{l},\ldots \right]}_{l=1,\ldots ,L_{x}}^{T}$$

Spans over the range grid $\left\{x_{l}\right\}$. The received signal $S_{m}$ can also be written as

(11) $$S_{m}=\Lambda_{m}F_{m}\alpha .$$

Since the target usually contains only a small number of dominant scattering centres relative to the total number of range resolution cells, the range profile α can be considered sparse (i.e., containing a small number of non-zero elements).

In the presence of unknown noise, (11) becomes

(12) $${\tilde{S}}_{m}=\Lambda_{m}F_{m}\alpha +n_{m}.$$

where $n_{m}$ is the additive noise for channel *m*. Stacking up the individual channel signals ${\tilde{S}}_{m}$, m=1,…,M, gives

(13) $$\tilde{S}=\Lambda F\alpha +n,$$

where

(14)S˜=[…,S˜mT,…]m=1,…,MT(15)Λ=diag{…,Λm,…}m=1,…,M(16)F=[…,FmT,…]m=1,…,MT(17)n=[…,nmT,…]m=1,…,MT.

Note that $\tilde{S}$ and n are column vectors of size $(\sum_{m}N_{m})\times 1$; diagonal phase error matrix Λ is of size $\left(\sum_{m}N_{m}\right)\times \left(\sum_{m}N_{m}\right)$; dictionary matrix F is $\left(\sum_{m}N_{m}\right)\times L_{x}$; and the range profile α is again a column vector of size $L_{x}\times 1$. Stacking the received signals amounts to a vertical stacking of the dictionary matrices from all channels and a diagonal concatenation of the corresponding phase error matrices. The stacking of multiple channels in this manner can improve the estimation accuracy for α, as will be demonstrated later in the paper.

A *problem statement* can thus be expressed as follows: given $\tilde{S}$ as the measured signal,

(18) $$\mathrm{find}\;\alpha \;\mathrm{and}\;\Lambda ,\;\mathrm{subject}\;\mathrm{to}\;\left\{\begin{array}{c} \tilde{S}\approx \Lambda F\alpha ,\\ \alpha \;\mathrm{is}\;\mathrm{sparse} \end{array}\right..$$

The estimation of α over $\left\{x_{l}\right\}$ is the process of range profiling, giving the main desired output, whereas the estimation of the phase error matrix Λ is really only a necessary intermediate result; it is a function of $\Delta R_{1},\Delta R_{2},\ldots ,$ and $\Delta R_{M}$ (recall that $\Delta R_{m}$ is the range estimation error resulting from the motion-compensation process for channel *m*). Furthermore, since these errors arise from a lack of precise knowledge of the relative locations of the radar channels’ phase centres and are small relative to a range resolution cell, we may assume, without loss of generality, that $\Delta R_{1}=0$.

## 3. Greedy Pursuit Solutions

In this section, we adopt the pruned OMP (POMP) technique, which was originally proposed for micro-Doppler parameter estimation, [24], for the problem of bandwidth stitching for range profiling. We start with the simplest case of two channels and then generalise it to the multiple channel case.

### 3.1. The Two-Channel Case

For this case, the signal model in (13) can be expressed as

(19) $$\tilde{S}=\Lambda \left(\Delta R_{2}\right)F\alpha +n,$$

where $\Lambda (\Delta R_{2})$ is a function of the single unknown relative range error $\Delta R_{2}$,

(20) $$\Lambda \left(\Delta R_{2}\right)=\mathrm{diag}\left\{I_{N_{1}},\Lambda_{2}\left(\Delta R_{2}\right)\right\},$$

with

(21) $$\Lambda_{2}\left(\Delta R_{2}\right)=\mathrm{diag}{\left\{\ldots ,\exp\left\{-\frac{4\pi jf_{2,n}}{c}\Delta R_{2}\right\},\ldots \right\}}_{n=1,\ldots ,N_{2}}.$$

In addition to the sparse range profile vector α, $\Delta R_{2}$ is the only additional unknown parameter to be estimated. Let us rewrite (19) as

(22) $$\tilde{S}=\Phi \left(\Delta R_{2}\right)\;\alpha +n$$

where

$$\Phi \left(\Delta R_{2}\right)=\Lambda \left(\Delta R_{2}\right)\;F.$$

In this form, the problem can be viewed as a joint sparse reconstruction and parameter estimation problem with the parametric dictionary $\Phi (\Delta R_{2})$ itself a function of the parameter $\Delta R_{2}$. This can be considered as a special dictionary learning problem where the objective is to solve simultaneously for both the sparse solution of α and the range error $\Delta R_{2}$.

To solve this problem, we adopt the POMP technique [24], which embeds a pruning operation into the iterative process of OMP. The main idea of POMP is to construct multiple realisations of the dictionary Φ based on a number $L_{\Delta }$ of candidate values of $\Delta R_{2}$; the OMP algorithm is applied to each dictionary realisation to find the atom which correlates most strongly with the current residual for that dictionary, and to recompute the residual with that atom’s contribution to the residual removed. To overcome possible excessive computations arising from outlier candidate values of $\Delta R_{2}$, a pruning operation is performed to exclude the half of the dictionaries which yield the largest residual errors, until a single dictionary realisation remains. The OMP iterations for the remaining dictionary are continued until a termination criterion is satisfied. The candidate value of $\Delta R_{2}$ corresponding to this dictionary gives the final estimate of $\Delta R_{2}$. The basic POMP algorithm is summarised in Table 1 and its computational cost is shown in Appendix C to be of the order of $L_{\Delta }N_{m}L_{x}$.

### 3.2. The Multi-Channel Case

For this case, the noisy signal model (19) becomes

(23) $$\tilde{S}=\Phi \left(\Delta R_{2},\ldots ,\Delta R_{M}\right)\alpha +n$$

where

(24) $$\Phi \left(\Delta R_{2},\ldots ,\Delta R_{M}\right)=\mathrm{diag}\left\{I_{N_{1}},\Lambda_{2}\left(\Delta R_{2}\right),\ldots ,\Lambda_{M}\left(\Delta R_{M}\right)\right\}\;F.$$

Here, the dictionary matrix is a function of the (M−1) unknowns $\Delta R_{2},\Delta R_{3},\ldots$ and $\Delta R_{M}$.

The POMP algorithm could be extended to multiple channels by computing candidate dictionaries based on a multi-dimensional grid of candidate values for $\Delta R_{2},\Delta R_{3},\ldots ,$ and $\Delta R_{M}$. The grid would consist of a total of (M−1) dimensions, where the *m*th dimension corresponds to the unknown range error $\Delta R_{m+1}$ of the (m+1) channel. Note that only a one-dimensional grid for $\Delta R_{2}$ is required for the case of two channels. However, the cardinality of the dictionary set is exponentially dependent on the number of available channels; i.e., $V^{\left(M-1\right)}$, where *V* denotes the number of grid points in each parameter dimension. As a result, although this extension would be simple and straightforward, it is computationally expensive.

To alleviate this computational burden, we instead apply the POMP algorithm pairwise to channels in order to estimate the range errors $\Delta R_{2},\ldots ,\Delta R_{M}$ relative to the first channel, and then utilise the conventional OMP algorithm to determine the range profile vector α based on these estimated values of $\Delta R_{2},\ldots ,\Delta R_{M}$. The procedure is summarised as below:

**STEP 1:** Estimation of range errors.

- For each pair between the 1^st^ and $m^{\mathrm{th}}$ channel, m∈{2,…,M}:
  - Calculate input signal:
    (25a) $$\begin{array}{cc} {\tilde{S}}_{1m} & ={\left[{\tilde{S}}_{1}^{T},{\tilde{S}}_{m}^{T}\right]}^{T} \end{array}$$
    (25b) $$\begin{array}{cc} F_{1m} & ={\left[F_{1}^{T},F_{m}^{T}\right]}^{T}. \end{array}$$
  - Construct candidate dictionaries based on a grid of *L* candidate values of $\Delta R_{m}^{\left(l\right)}$ (l=1,…,L):
    (25c) $$\Phi_{1m,l}=\mathrm{diag}\left\{I_{N_{1}},\Lambda_{m}\left(\Delta R_{m}^{\left(l\right)}\right)\right\}F_{1m}.$$
  - Perform POMP given ${\tilde{S}}_{1m}$ and $\Phi_{1m,1},\ldots ,\Phi_{1m,L}$ to obtain an estimate of $\Delta R_{m}$ (denoted as ${\hat{\Delta R}}_{m}$).

 End for.

**STEP 2:** Estimation of range profile vector.

- Compute signal and dictionary.
  (26)S˜=[…,S˜mT,…]m=1,…,MT(27)F=[…,FmT,…]m=1,…,MT(28)Λ=diag{…,Λm(ΔR^m),…}m=1,…,M(29)Φ=ΛF.
- Estimate α using OMP given $\tilde{S}$ and Φ.

The computational cost of the general POMP algorithm is shown in Appendix C to be of the order of $\left(M-1\right)L_{\Delta }N_{m}L_{x}$.

## 4. $L_{1}$-Norm Regularisation Approach

The sparse reconstruction problem (18) can be solved via the following $l_{1}$ regularised optimisation:

(30) $$\min_{\alpha ,\Lambda }\left\{{∥S-\Lambda F\alpha ∥}_{2}^{2}+\mu {∥\alpha ∥}_{1}\right\},$$

where μ is a regularisation parameter. It should be emphasized that this is not a conventional $l_{1}$ regularisation formulation because of the unknown phase error matrix Λ resulting from the estimation error of the motion-compensation phase. Therefore, Λ must be jointly estimated with α:

(31) $$\left\{\hat{\alpha },\hat{\Lambda }\right\}=\underset{\alpha ,\Lambda }{arg min}\left\{{∥S-\Lambda F\alpha ∥}_{2}^{2}+\mu {∥\alpha ∥}_{1}\right\}.$$

Two solutions for this joint estimation problem were presented in [25]. The first solution assumes that the phase error matrix for the $m^{\mathrm{th}}$ sub-band is modelled as

(32) $$\Lambda_{m}=\exp\left\{j\phi_{m}\right\}\;I_{N_{m}\times N_{m}}.$$

In other words, the phase errors for different frequency bins of a particular sub-band are identical. However, this assumption is invalid in the problem under consideration because the phase error is a function of frequency and thus has different values for different frequency bins. Therefore, that solution is not applicable in this case. The second solution considers a general phase error matrix

(33) $$\Lambda_{m}=\mathrm{diag}{\left\{\ldots ,\exp\left\{j\phi_{m,n}\right\},\ldots \right\}}_{n=1,\ldots ,N_{m}}$$

where the phase errors $\phi_{m,n}$ can be arbitrary. Although this solution can be used, it does not exploit the underlying structure of the phase errors; i.e., $\phi_{m,n}=-\frac{4\pi f_{m,n}}{c}\Delta R_{m}$. In what follows, we will also present other refined versions, building on the second solution of [25], while exploiting prior knowledge of the structure of the phase error.

The $l_{1}$ norm can be approximated as [26,27,28,29]:

(34) $${∥\alpha ∥}_{1}\approx \sum_{l=1}^{L}{\left(|\alpha_{l}{|}^{2}+\delta \right)}^{1/2}$$

In order to overcome the nondifferentiably of the $l_{1}$ norm at the origin. Here, δ is a small non-negative parameter. Using this approximation, the minimisation problem in (31) becomes

(35) $$\left\{\hat{\alpha },\hat{\Lambda }\right\}=\underset{\alpha ,\Lambda }{arg min}\left\{{∥S-\Lambda F\alpha ∥}_{2}^{2}+\lambda \sum_{l=1}^{L}{\left(|\alpha_{l}{|}^{2}+\delta \right)}^{1/2}\right\}.$$

The solution of (35) tends to the solution of (31) as δ approaches zero. Therefore, a small value of δ should be used to ensure the validity of this approximation. The quasi-Newton approach can be adopted to solve the modified $l_{1}$ regularised optimisation (35), as below.

The gradient of the objective function of (35) is given by

(36) $$\nabla \left(\alpha \right)=H\left(\alpha \right)\alpha -2\;F^{H}\Lambda^{H}S,$$

where the superscript ${}^{H}$ denotes the Hermitian transpose operation. Here, H is the Hessian matrix given by

(37) $$H\left(\alpha \right)=2\;F^{H}\Lambda^{H}\Lambda F+\lambda W\left(\alpha \right)=2F^{H}F+\lambda W\left(\alpha \right),$$

where

(38) $$W\left(\alpha \right)=\mathrm{diag}\left\{\ldots ,{\left(|\alpha_{l}{|}^{2}+\delta \right)}^{-1/2},\ldots \right\}.$$

Since the Hessian matrix is a function of the unknown α, the minimisation (35) is solved iteratively. Given the estimates $\hat{\alpha }\left(i\right)$ and $\hat{\Lambda }\left(i\right)$ from a previous iteration *i*, the new solutions at iteration i+1 are obtained in the following two steps.

1. Calculate $\hat{\alpha }\left(i+1\right)$ by setting ∇(α)=0 given $H(\hat{\alpha }\left(i\right))$ and $\hat{\Lambda }\left(i\right)$:
  (39) $$\begin{array}{cc} \hat{\alpha }\left(i+1\right) & =2\;{\left(H\left(\hat{\alpha }\left(i\right)\right)\right)}^{-1}F^{H}{\left(\hat{\Lambda }\left(i\right)\right)}^{H}S\\ \; & ={\left(F^{H}F+\frac{1}{2}\lambda W\left(\hat{\alpha }\left(i\right)\right)\right)}^{-1}F^{H}{\left(\hat{\Lambda }\left(i\right)\right)}^{H}S. \end{array}$$
2. Calculate $\hat{\Lambda }\left(i+1\right)$ given $\hat{\alpha }\left(i+1\right)$. The phase error matrix $\hat{\Lambda }\left(i+1\right)$ is obtained by solving:
  (40) $$\hat{\Lambda }\left(i+1\right)=\underset{\Lambda }{arg min}{∥S-\Lambda F\hat{\alpha }\left(i+1\right)∥}_{2}^{2}$$
  or equivalently
  (41) $${\hat{\Lambda }}_{m}\left(i+1\right)=\underset{\Lambda_{m}}{arg min}{∥S_{m}-\Lambda_{m}F_{m}\hat{\alpha }\left(i+1\right)∥}_{2}^{2}$$
  for m=2,…,M. Note that $\Lambda_{1}=I_{N_{1}}$ (since $\Delta R_{1}=0$); thus, no estimation is required for $\Lambda_{1}$.

The algorithm may be halted when the objective function falls below a threshold, or when a maximum number of iterations is reached, or when the relative change in the objective function falls below a threshold.

Various methods for calculating the phase error matrix $\hat{\Lambda }\left(i+1\right)$ in Step 2 are given in the following sections.

### 4.1. Unstructured Approach

Ignoring the underlying structure of the phase errors, i.e., $\phi_{m,n}=-\frac{4\pi f_{m,n}}{c}\Delta R_{m}$, ${\hat{\Lambda }}_{m}$ can be considered as a diagonal matrix with arbitrary elements $\phi_{m,n}$:

(42) $$\Lambda_{m}=\mathrm{diag}{\left\{\ldots ,\exp\left\{j\phi_{m,n}\right\},\ldots \right\}}_{n=1,\ldots ,N_{m}}.$$

Therefore, $\phi_{m,n}$ can be estimated as [25]

(43) $${\hat{\phi }}_{m,n}\left(i+1\right)=\tan^{-1}\frac{ℑ\{S_{m,n}{\hat{Y}}_{m,n}^{*}\left(i+1\right)\}}{ℜ\{S_{m,n}{\hat{Y}}_{m,n}^{*}\left(i+1\right)\}}$$

where ℑ{·} and ℜ{·} denote operations to extract the imaginary and real parts of a complex number, and $\tan^{-1}$ stands for a four-quadrant arctangent operation. Here, ${\hat{Y}}_{m,n}\left(i+1\right)$ is the $n^{\mathrm{th}}$ element of ${\hat{Y}}_{m}\left(i+1\right)$ which is defined as ${\hat{Y}}_{m}\left(i+1\right)=F_{m}\hat{\alpha }\left(i+1\right)$. As a result, we obtain:

(44) $${\hat{\Lambda }}_{m}\left(i+1\right)=\mathrm{diag}{\left\{\ldots ,\exp\left\{j{\hat{\phi }}_{m,n}\left(i+1\right)\right\},\ldots \right\}}_{n=1,\ldots ,N_{m}}.$$

### 4.2. Gauss–Newton Approach

Taking into account the underlying structure of the phase errors, $\Lambda_{m}\left(\Delta R_{m}\right)$ is a function of $\Delta R_{m}$, and the minimisation (41) can be re-expressed as

(45) $${\hat{\Delta R}}_{m}\left(i+1\right)=\underset{\Delta R_{m}}{arg min}{∥S_{m}-\Lambda_{m}\left(\Delta R_{m}\right)F_{m}\hat{\alpha }\left(i+1\right)∥}_{2}^{2}.$$

By letting $e_{m}=S_{m}-\Lambda_{m}\left(\Delta R_{m}\right)F_{m}\hat{\alpha }\left(i+1\right)$, we have

(46) $$e_{m}={\left[\ldots ,e_{m,n},\ldots \right]}_{n=1,\ldots ,N_{m}}^{T}$$

where

(47) $$e_{m,n}=S_{m,n}-U_{m,n}\exp\left\{-\frac{4\pi jf_{m,n}}{c}\Delta R_{m}\right\}\sum_{l=1}^{L}{\hat{\alpha }}_{l}\left(i+1\right)\exp\left\{-\frac{4\pi jf_{m,n}}{c}x_{l}\right\}$$

and

(48) $$U_{m,n}=\exp\left\{j\phi_{m,n}\right\}.$$

Here ${\hat{\alpha }}_{l}\left(i+1\right)$ is the $l^{\mathrm{th}}$ element of $\hat{\alpha }\left(i+1\right)$. As we are estimating real quantities, it is more convenient to reformulate the problem as the minimisation of a real function in order to apply the Gauss–Newton. The details of the Gauss–Newton algorithm for updating ${\hat{\Delta R}}_{m}\left(i+1\right)$ are given in Appendix A.

Using ${\hat{\Delta R}}_{m}\left(i+1\right)$, we obtain the phase error matrix as

(49) $${\hat{\Lambda }}_{m}\left(i+1\right)=\mathrm{diag}{\left\{\ldots ,\exp\left\{-\frac{4\pi j{\hat{\Delta R}}_{m}\left(i+1\right)}{c}f_{m,n}\right\},\ldots \right\}}_{n=1,\ldots ,N_{m}}.$$

### 4.3. Linear Regression-Based Approach

By noting that $\phi_{m,n}=-\frac{4\pi \Delta R_{m}}{c}f_{m,n}$, the gradient $-\frac{4\pi \Delta R_{m}}{c}$ can be calculated via a linear least squares estimator using ${\hat{\phi }}_{m,n}$ obtained from (43) [30]. Specifically, we have

(50) $${\left[-\frac{4\pi {\hat{\Delta R}}_{m}\left(i+1\right)}{c},{\hat{\phi }}_{m}^{†}\left(i+1\right)\right]}^{T}={\left(A_{m}^{T}A_{m}\right)}^{-1}A_{m}^{T}b_{m}\left(i+1\right)$$

where

(51a)Am=[…,Am,n,…]n=1,…,NmT,withAm,n=[fm,n,1](51b)bm(i+1)=[…,ϕ^m,nunwrapped(i+1),…]n=1,…,NmT.

Note that ${\hat{\phi }}_{m,n}^{\mathrm{unwrapped}}\left(i+1\right)$ is the unwrapped version of ${\hat{\phi }}_{m,n}\left(i+1\right)$ and ${\hat{\phi }}_{m}^{†}\left(i+1\right)$ is an estimate for the initial phase $\phi_{m}^{†}\left(i+1\right)$ which results from the unwrapping process. From (50), ${\hat{\Delta R}}_{m}\left(i+1\right)$ is obtained and can then be used for computing ${\hat{\Lambda }}_{m}\left(i+1\right)$ as in (49).

### 4.4. Differenced-Phase-Based Approach

Subtracting the estimated phase errors of two successive frequency bins, we obtain:

(52) $$-\frac{4\pi (f_{m,n+1}-f_{m,n})}{c}\Delta R_{m}=\phi_{m,n+1}-\phi_{m,n}.$$

Therefore, $\Delta R_{m}$ can be estimated as [30]

(53) $${\hat{\Delta R}}_{m}\left(i+1\right)=-\frac{c}{4\Delta f_{m}\left(N_{m}-1\right)}\sum_{n=1}^{N_{m}-1}\Delta \phi_{m,n}\left(i+1\right)$$

where

(54) $$\Delta \phi_{m,n}\left(i+1\right)=\tan^{-1}\frac{\sin({\hat{\phi }}_{m,n+1}\left(i+1\right)-{\hat{\phi }}_{m,n}\left(i+1\right))}{\cos({\hat{\phi }}_{m,n+1}\left(i+1\right)-{\hat{\phi }}_{m,n}\left(i+1\right))}.$$

Note that the four-quadrant arctangent has been used here to handle the phase wrapping. An estimate of the phase error matrix ${\hat{\Lambda }}_{m}\left(i+1\right)$ is now obtained as in (49) using ${\hat{\Delta R}}_{m}\left(i+1\right)$ in (53).

## 5. Simulation and Discussion

Numerical simulations are presented in this section to evaluate the performance of the methods described in previous sections.

### 5.1. Scenario 1: Two Sub-Bands

**Figure 1:**
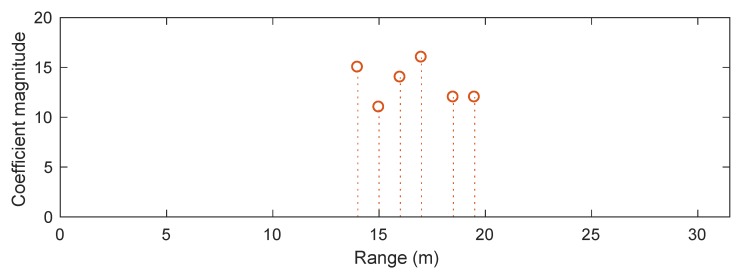
True range profile of synthetic target under consideration.

We consider a synthetic scenario with two sub-bands at carrier frequencies of $f_{1}=6$ GHz and $f_{2}=8$ GHz, each having a bandwidth of B=300 MHz and 64 frequency steps (i.e., $N=N_{1}=N_{2}=64$). The range profile is discretised over a grid with a length of (N−1)c/(2B)=31.5 m and a grid step of $\Delta_{\mathrm{Grid}}=c/\left(10B\right)=0.1$ m. We consider a far-field target consisting of six point scatterers which are aligned with the grid. Figure 1 plots the true range profile of the target. We set $\Delta R_{2}=2.78\Delta_{\mathrm{Grid}}$ for the case of existing phase errors. The signal-to-noise ratio is set to 10 dB.

**Figure 2:**
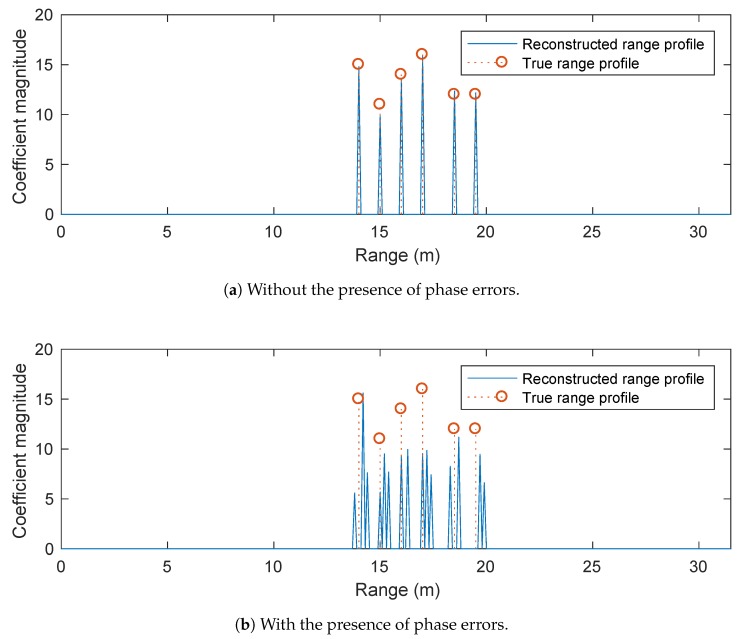
Performance of conventional OMP.

**Figure 3:**
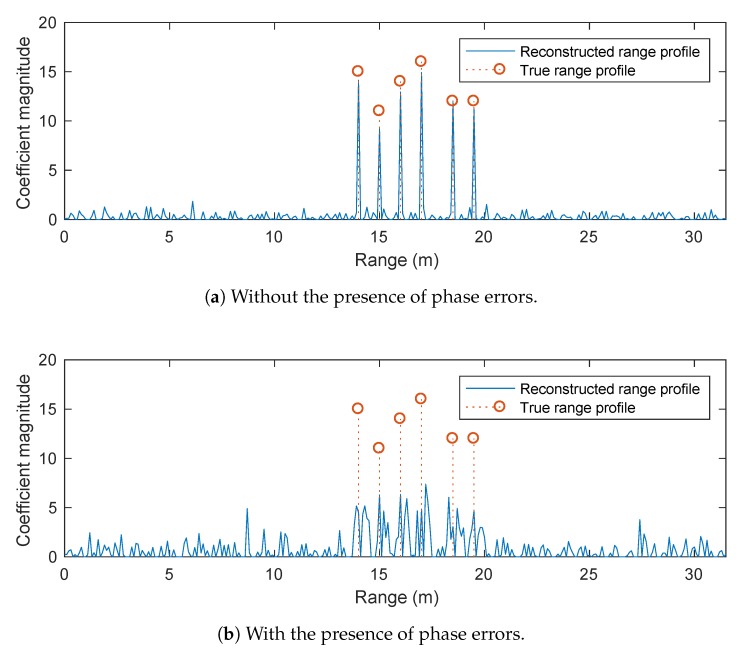
Performance of conventional $l_{1}$-norm regularised optimisation solver.

Figure 2 compares the reconstructed range profiles obtained by the conventional OMP algorithm without and with the presence of phase errors. The OMP is terminated when the signal residual reaches the noise level or after 15 iterations have been carried out. We observe that OMP successfully reconstructs the range profile of the target by correctly identifying the scatterers of the target with accurate range and coefficient estimates when no phase errors exist. However, OMP provides unsatisfactory results in the presence of phase errors, where the reconstructed image is observed as exhibiting many spurious scatterers. Similar observations are obtained for the results obtained by the conventional $l_{1}$-norm regularised optimisation solver (without phase error correction), as shown in Figure 3. Here, we set $\delta =10^{-5}$ and $\lambda =0.001\max|c_{l}|$ where $c_{l}$ is the $l^{\mathrm{th}}$ element of $c=F^{H}\tilde{S}$. The $l_{1}$-norm regularised optimisation solver is stopped if the relative change in the $l_{2}$-norm of the range profile vector α falls below $10^{-5}$ or after it reaches 500 iterations. The performance degradation of these conventional sparse reconstruction techniques is not unexpected, since they were not originally developed to cope with dictionary mismatch arising from the presence of the phase errors.

**Figure 4:**
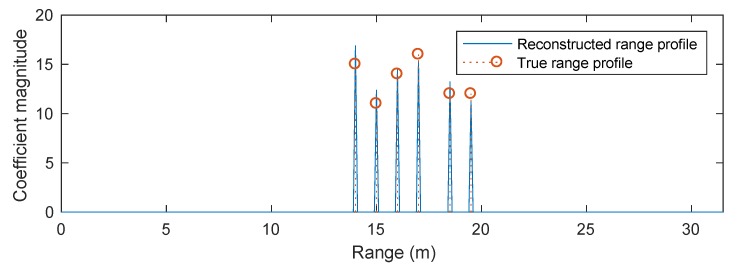
Performance of POMP in the presence of phase errors.

Figure 4 shows the reconstructed range profile obtained by the POMP. POMP constructs candidate dictionaries based on a grid of $\Delta R_{2}$ with a grid step size of $\Delta_{\mathrm{Grid}}/100$. The same stopping criteria of OMP is used for POMP. We observe that POMP produces a range profile which is almost identical to the ground truth, thereby demonstrating the effectiveness of POMP in terms of dealing with the phase errors between different sub-bands.

**Figure 5:**
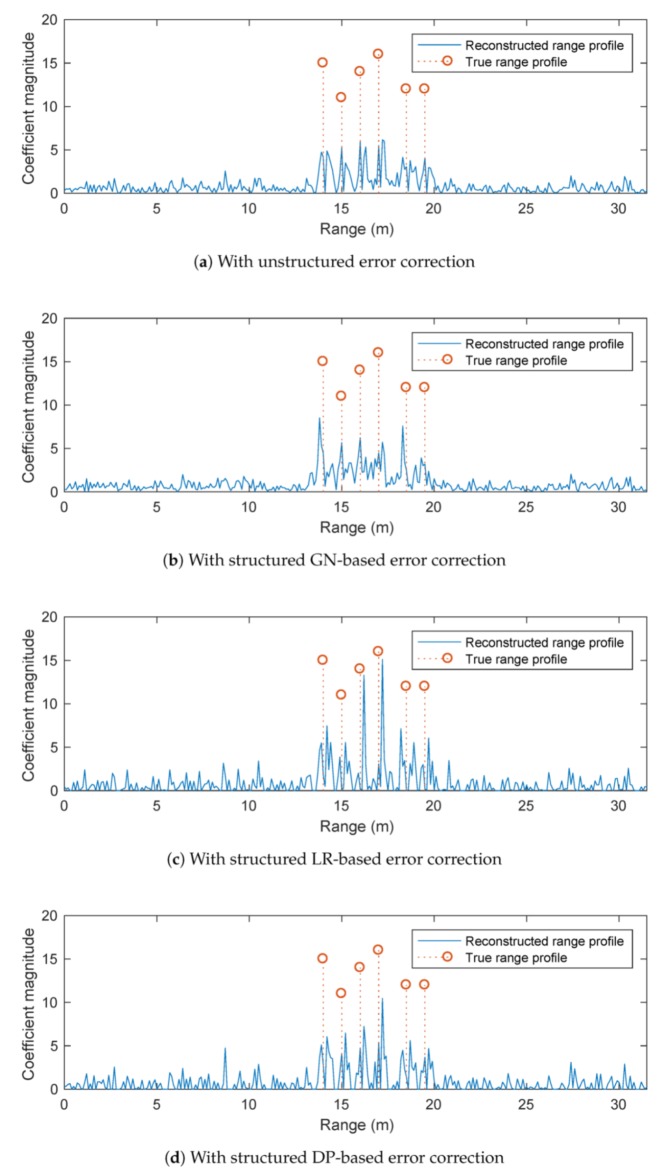
Performance of $l_{1}$-norm regularised optimisation solver with phase error correction.

Figure 5 shows the results obtained by different $l_{1}$-norm regularised optimisation solvers with phase error correction, as presented in Section 4. The same parameters and stopping criteria of the conventional $l_{1}$-norm regularised optimisation solver as described above are used in the simulations. Although these algorithms exhibit some improvements over the conventional $l_{1}$-norm regularisation (i.e., without phase error correction), they provide poorer results compared to that of POMP. Specifically, the peaks of the reconstructed range profiles obtained by these algorithms only appear close to but not exactly at the true scatterer positions. In addition, the magnitudes of the peaks are much smaller than the ground truth values.

**Figure 6:**
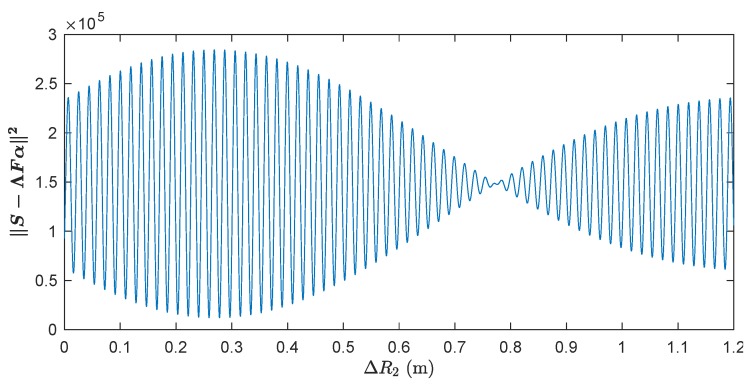
Illustration of the nonconvexity of the $l_{1}$-norm regularised optimisation problem (31). The objective function of (31) is plotted against $\Delta R_{2}$ assuming that α is perfectly known.

The inferior performances of these algorithms can be explained by noting that the $l_{1}$-norm regularised optimisation in (31) is a nonconvex problem due to the phase error matrix Λ. Figure 6 plots the objective function of (31) as a function of $\Delta R_{2}$ assuming that α is perfectly known. We observe that this objective function has many local maxima and minima, confirming the non-convexity of the $l_{1}$-norm regularised optimisation in (31). The reason for this non-convexity is explained in Appendix B. Due to this nonconvexity, the iterative solvers presented in Section 4 are prone to converge to local minima; thus, limiting the effectiveness of this approach.

**Figure 7:**
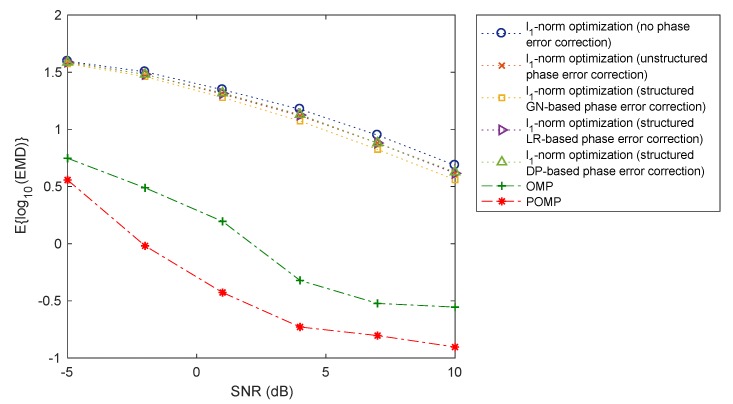
Earth mover’s distance (EMD) performance of the OMP, POMP, and $l_{1}$-norm regularised optimisation methods versus various of SNRs ($\Delta R_{2}=2.78\Delta_{\mathrm{Grid}}$).

**Figure 8:**
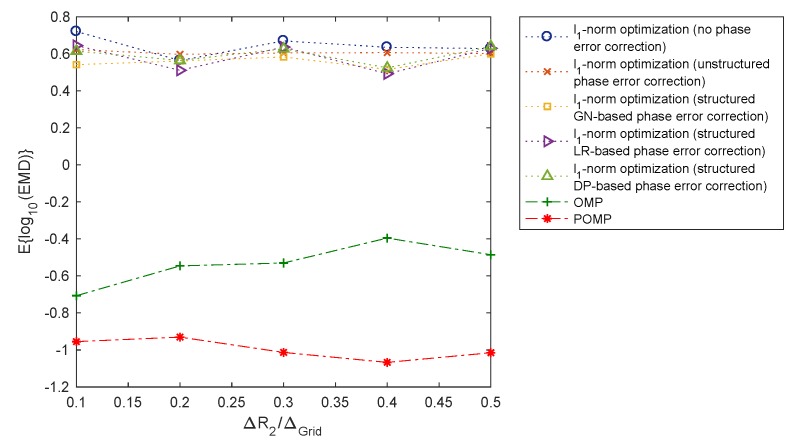
EMD performance of the OMP, POMP, and $l_{1}$-norm regularised optimisation methods versus various levels of phase error (SNR=10 dB).

The performance of the OMP, POMP, and $l_{1}$-norm regularised optimisation methods are now compared using the earth mover’s distance (EMD) between the true and reconstructed range profiles. EMD [31] is a metric estimating the distance between two distributions or equivalently the minimal amount of work required to transform one distribution to the other. Figure 7 and Figure 8 show the EMD performance of the OMP, POMP, and $l_{1}$-norm regularised optimisation methods, averaged over 100 Monte Carlo runs, versus different levels of SNR (signal-to-noise ratio) and phase error, respectively. It is observed that the POMP method yields the smallest EMD values amongst all algorithms considered. Since a smaller value of EMD corresponds to a higher level of similarity between the true and reconstructed range profiles, this observation indicates that the reconstructed range profile obtained by POMP is closer to the ground-truth range profiles than those obtained from the OMP and $l_{1}$-norm regularised optimisation methods. This verifies the performance advantage of the POMP method from a statistical point of view.

### 5.2. Scenario 2: Four Sub-Bands

We now consider another scenario with four sub-bands at carrier frequencies of $f_{1}=6$ GHz, $f_{2}=8$ GHz, $f_{3}=10$ GHz, and $f_{4}=12$ GHz, each having a bandwidth of B=300 MHz and 64 frequency steps (i.e., $N=N_{1}=N_{2}=N_{3}=N_{4}=64$). The range errors are set to $\Delta R_{2}=2.78\Delta_{\mathrm{Grid}}$, $\Delta R_{3}=1.33\Delta_{\mathrm{Grid}}$, and $\Delta R_{4}=3.69\Delta_{\mathrm{Grid}}$. Other simulation parameters and the true range profile of the target remain unchanged as in the previous simulation example.

**Figure 9:**
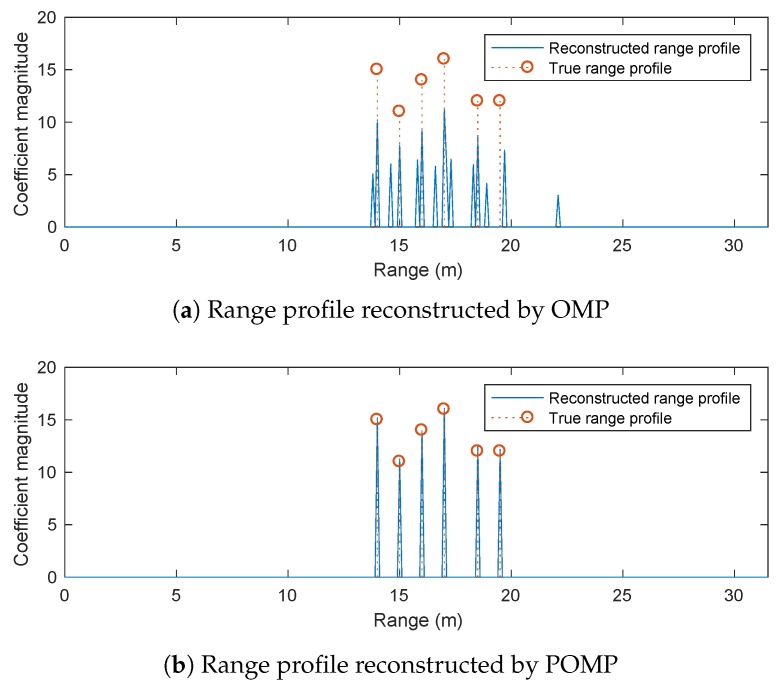
Performance comparison between OMP and POMP for Simulation Scenario 2 (with four sub-bands).

Figure 9 compares the reconstructed range profiles obtained by the conventional OMP algorithm and the POMP algorithm presented in Section 3.2. OMP results in an unsatisfactorily reconstructed range profile with many spurious peaks, as expected, because it ignores the phase errors between different sub-bands. In contrast, the POMP is capable of reconstructing the true range profile with a high accuracy thanks to the use of dictionary learning with a pruning process. Note that, given the inferior performance of the $l_{1}$-norm regularised optimisation approach compared to POMP, as demonstrated in the previous simulation scenario, this approach is excluded from the comparison here.

## 6. Conclusions

This paper explores the use of the POMP algorithm and $l_{1}$-norm regularisation solvers for the problem of sparsity-driven HRRP with bandwidth stitching in the presence of phase errors. We observe that the $l_{1}$-norm regularisation solvers do not provide significant performance improvement over the conventional sparse reconstruction algorithms due to the nonconvexity of $l_{1}$-norm regularised optimisation when phase errors exists. In contrast, POMP is observed to be capable of effectively dealing with the phase errors and thus be able to reconstruct the range profile of the target with high accuracy. Simulation results show a significant performance improvement by POMP over OMP and the conventional and refined $l_{1}$-norm regularisation. In future work, we propose using experimental data for a more general scenario where the true scatterers constituting the target are located in off-grid positions with respect to the dictionary grid, and the true range errors have off-grid values. We shall also consider the more general case of frequency-dependence of scatterer RCS (radar cross-section). A potential approach for this is to exploit the framework of spectral compressive sensing [32,33].

## Figures and Tables

**Table 1:** The pruned orthogonal matching pursuit (POMP) algorithm (*M* = 2).

INPUT: Noisy signal data vector $\tilde{S}$ Candidate dictionaries $\Phi_{1},\Phi_{2},\ldots ,\Phi_{L_{\Delta }}$, corresponding to $L_{\Delta }$ candidate values of $\Delta R_{2}$ PROCEDURE: Initialization: - set the initial indexes of active dictionaries to $\Theta_{1}=\left\{1,\ldots ,L_{\Delta }\right\}$; - set the corresponding residual vectors to $r_{1}=\cdots =r_{L_{\Delta }}=\tilde{S}$; - set the initial support Λ to ∅, the null set; **for**i=1; i:=i+1 until $|\Theta_{i}|==1$ (the cardinality of $\Theta_{i}$) and $|r_{l}|<\epsilon$ for $l\in \Theta_{i}$, **for** every $l\in \Theta_{i}$, perform OMP as follows - Identify: $c_{l}=\Phi_{l}^{H}r_{l}$ $j_{l}={arg max}_{j}|c_{j}|$ - Merge supports: $\Lambda_{l}=\Lambda_{l}\cup j_{l}$ - Update${}^{*}$: ${\hat{\alpha }}_{l,\Lambda_{l}}={\left(\Phi_{l,\Lambda_{l}}^{H}\Phi_{l,\Lambda_{l}}\right)}^{-1}\Phi_{l,\Lambda_{l}}^{H}\tilde{S}$ $r_{l}=\tilde{S}-\Phi_{l,\Lambda_{l}}{\hat{\alpha }}_{l,\Lambda_{l}}$ **end for** **if**$|\Theta_{i}|>1$ Remove indices of $\left\lceil |\Theta_{i}|/2\right\rceil$ candidate dictionaries that correspond to $\left\lceil |\Theta_{i}|/2\right\rceil$ largest residual errors from $|\Theta_{i}|$ **end if** **end for** OUTPUT: The range profile estimate ${\hat{\alpha }}_{l^{★},\Lambda_{l^{★}}}$ where $l^{★}$ is the last remaining element of $\Theta_{i}$ The estimate of $\Delta R_{2}$ is the value of $\Delta R_{2}$ corresponding to $\Phi_{l^{★}}$

${}^{*}$$\Phi_{l,\Lambda_{l}}$ consists of the columns of $\Phi_{l}$ with indices belonging to $\Lambda_{l}$ and ${\hat{\alpha }}_{\Lambda_{l}}$ consists of the elements of ${\hat{\alpha }}_{l}$ with indices belonging to $\Lambda_{l}$.

## Appendix A. Derivation of Gauss–Newton Algorithm for Estimation of ${\hat{\Delta \;R}}_{m}\left(i+1\right)$

We rewrite $e_{m,n}$ as

(A1) $$e_{m,n}=S_{m,n}-\exp\left\{-\frac{4\pi jf_{m,n}}{c}\Delta R_{m}\right\}Z_{m,n}$$

with

(A2) $$Z_{m,n}=U_{m,n}\sum_{l=1}^{L}{\hat{\alpha }}_{l}\left(i+1\right)\exp\left\{-\frac{4\pi jf_{m,n}}{c}x_{l}\right\}.$$

By noting that

(A3) $$\begin{array}{cc} \; & \exp\left\{-\frac{4\pi jf_{m,n}}{c}\Delta R_{m}\right\}Z_{m,n}\\ \; & =Z_{m,n}^{R}\cos\left\{\frac{4\pi f_{m,n}}{c}\Delta R_{m}\right\}+Z_{m,n}^{I}\sin\left\{\frac{4\pi f_{m,n}}{c}\Delta R_{m}\right\}\\ \; & \;+j\left(Z_{m,n}^{I}\cos\left\{\frac{4\pi f_{m,n}}{c}\Delta R_{m}\right\}-Z_{m,n}^{R}\sin\left\{\frac{4\pi f_{m,n}}{c}\Delta R_{m}\right\}\right) \end{array}$$

where $Z_{m,n}^{R}$ and $Z_{m,n}^{I}$ are the real and imaginary components of $Z_{m,n}$, we can decouple and stack the real and imaginary components of $e_{m}$ to form a real-valued vector as

(A4) $$\epsilon_{m}={\left[{\left(e_{m}^{R}\right)}^{T},{\left(e_{m}^{I}\right)}^{T}\right]}^{T}$$

where

(A5) $$\begin{array}{c} e_{m}^{R}={\left[\ldots ,e_{m,n}^{R},\ldots \right]}_{n=1,\ldots ,N_{m}}^{T} \end{array}$$

(A6) $$\begin{array}{c} e_{m}^{I}={\left[\ldots ,e_{m,n}^{I},\ldots \right]}_{n=1,\ldots ,N_{m}}^{T} \end{array}$$

and

(A7) $$\begin{array}{cc} e_{m,n}^{R} & =Z_{m,n}^{R}\cos\left\{\frac{4\pi f_{m,n}}{c}\Delta R_{m}\right\}+Z_{m,n}^{I}\sin\left\{\frac{4\pi f_{m,n}}{c}\Delta R_{m}\right\} \end{array}$$

(A8) $$\begin{array}{cc} e_{m,n}^{I} & =Z_{m,n}^{I}\cos\left\{\frac{4\pi f_{m,n}}{c}\Delta R_{m}\right\}-Z_{m,n}^{R}\sin\left\{\frac{4\pi f_{m,n}}{c}\Delta R_{m}\right\}. \end{array}$$

Using (46)–(A8), the minimization (45) becomes

(A9) $${\hat{\Delta R}}_{m}\left(i+1\right)=\underset{\Delta R_{m}}{arg min}{∥\epsilon \left(\Delta R_{m}\right)∥}_{2}^{2}.$$

By adopting the Gauss–Newton algorithm, ${\hat{\Delta R}}_{m}\left(i+1\right)$ can be computed from ${\hat{\Delta R}}_{m}\left(i\right)$ as

(A10) $${\hat{\Delta R}}_{m}\left(i+1\right)={\hat{\Delta R}}_{m}\left(i\right)-{\left(J_{m}^{T}\left(i\right)J_{m}\left(i\right)\right)}^{-1}J_{m}^{T}\left(i\right)\epsilon \left({\hat{\Delta R}}_{m}\left(i\right)\right)$$

where $\epsilon ({\hat{\Delta R}}_{m}\left(i\right))$ is an estimated version of ϵ computed from ${\hat{\Delta R}}_{m}\left(i\right)$ and $J_{m}\left(i\right)$ is the Jacobian of $\epsilon (\Delta R_{m})$ with respect to $\Delta R_{m}$ evaluated at $\Delta R_{m}={\hat{\Delta R}}_{m}\left(i\right)$.

The expression for the Jacobian $J_{m}$ of $\epsilon (\Delta R_{m})$ with respect to $\Delta R_{m}$ is given by

(A11) $$J_{m}={\left[{\left(J_{m}^{R}\right)}^{T},{\left(J_{m}^{I}\right)}^{T}\right]}^{T}$$

where

(A12) $$\begin{array}{cc} J_{m}^{R} & ={\left[\ldots ,J_{m,n}^{R},\ldots \right]}_{n=1,\ldots ,N_{m}}^{T} \end{array}$$

(A13) $$\begin{array}{cc} J_{m}^{I} & ={\left[\ldots ,J_{m,n}^{I},\ldots \right]}_{n=1,\ldots ,N_{m}}^{T} \end{array}$$

and

(A14) $$\begin{array}{cc} J_{m,n}^{R} & =\frac{4\pi f_{m,n}}{c}\left(-Z_{m,n}^{R}\sin\left\{\frac{4\pi f_{m,n}}{c}\Delta R_{m}\right\}+Z_{m,n}^{I}\cos\left\{\frac{4\pi f_{m,n}}{c}\Delta R_{m}\right\}\right) \end{array}$$

(A15) $$\begin{array}{cc} J_{m,n}^{I} & =-\frac{4\pi f_{m,n}}{c}\left(Z_{m,n}^{I}\sin\left\{\frac{4\pi f_{m,n}}{c}\Delta R_{m}\right\}+Z_{m,n}^{R}\cos\left\{\frac{4\pi f_{m,n}}{c}\Delta R_{m}\right\}\right). \end{array}$$

## Appendix B. Analysis of the Nonconvexity of the $l_{1}$-Norm Regularised Optimisation Problem **(31)**

From Equation (31)

(A16) $$\left\{\hat{\alpha },\hat{\Lambda }\right\}=\underset{\alpha ,\Lambda }{arg min}\left\{{∥S-\Lambda F\alpha ∥}_{2}^{2}+\mu {∥\alpha ∥}_{1}\right\},$$

or

(A17) $$\left\{\hat{\alpha },\hat{\Lambda }\right\}=\underset{\alpha }{arg min}\underset{\Lambda }{arg min}\left\{{∥S-\Lambda F\alpha ∥}_{2}^{2}+\mu {∥\alpha ∥}_{1}\right\}.$$

(A18) $${∥S-\Lambda F\alpha ∥}_{2}^{2}={\left\{S-\Lambda F\alpha \right\}}^{H}\left\{S-\Lambda F\alpha \right\}$$

(A19) $${∥S-\Lambda F\alpha ∥}_{2}^{2}=S^{H}S-2ℜe\left[{\left(F^{H}\Lambda^{H}S\right)}^{H}\alpha \right]+\alpha^{H}F^{H}F\alpha .$$

Only the term ${\left(F^{H}\Lambda^{H}S\right)}^{H}\alpha$ is dependant on $\Delta R_{2}$, so the objective function is minimised with respect to $\Delta R_{2}$ when the correlation between $F^{H}\Lambda^{H}S$ and α is maximised. Now

(A20) $$F^{H}\Lambda^{H}S=F_{1}^{H}S_{1}+F_{2}^{H}\Lambda_{2}^{H}S_{2}$$

And the terms $F_{1}^{H}S_{1}$ and $F_{2}^{H}\Lambda_{2}^{H}S_{2}$ represent the conventional pulse compression (i.e., transformation from frequency domain to range domain) for each of the radars with a range offset $\Delta R_{2}$. These are non-sparse and low resolution due to the oversampling in range. So $F^{H}\Lambda^{H}\left(\Delta R_{2}\right)S$ represents the linear superposition of the two conventional range profiles. When these range profiles from the two radars are correctly aligned in range, they will better correlate with the true range profile α. Also if the range offset $\Delta R_{2}$ is such that a scatterer for one radar is superimposed upon a different scatterer for the other radar, a local minimum will occur. That explains Figure 6 and the reason for its non-convexity.

## Appendix C. Analysis of Operation Count for POMP

Consider first the case of M=2. Let the size of the grid for the parameter $\Delta R_{2}$ be $L_{\Delta }=2^{N_{\Delta }}$, so that a dictionary is constructed for each of these $2^{N_{\Delta }}$ values of $\Delta R_{2}$. OMP is implemented with the number of dictionaries halved at each stage; hence, the name “pruned” OMP. At stage *k* of OMP, the number of complex multiplications and divisions for a single dictionary is denoted $C_{omp}\left(k\right)$. Here $k=1,\ldots ,N_{\Delta }+1$ with $2^{N_{\Delta }-k+1}$ dictionaries considered at stage *k*. The purpose of this section is to estimate the dependence of the computational cost of POMP on the size of the dictionary $L_{x}$ and the grid size $L_{\Delta }$ for the parameter $\Delta R_{2}$.

With reference to Table 1, the significant costs for POMP are associated with the Identify and Update steps. At each stage of OMP for a given dictionary, the Identify step performs atom/residual correlations which require $\;∼O(N_{m}L_{x})$ complex multiplications. The Update step performs a linear least squares estimation requiring Gaussian elimination which, at stage *k* of OMP, has an operation count $∼O(k^{3})$.

Due to the halving of the number of dictionaries at each stage, the total operational count required until only one dictionary is left (although more OMP steps may be required for that dictionary until the residual is sufficiently small) is therefore of order

(A21) $$\sum_{k=1}^{N_{\Delta }+1}\left(N_{m}L_{x}+k^{3}\right)2^{N_{\Delta }-k+1}$$

or

(A22) $$\left(2^{N_{\Delta }+1}-1\right)N_{m}L_{x}+{\left(N_{\Delta }+1\right)}^{3}+2^{N_{\Delta }+1}\sum_{k=1}^{N_{\Delta }}k^{3}z^{k}$$

with $z=\frac{1}{2}$.

The finite sum $\sum_{k=1}^{n}k^{3}z^{k}$ is referred to as a low-order polylogarithm, for which a formula may be derived [34]. This formula can be shown to have a leading term of order $n^{3}z^{n+3}$ so that the overall operation count for POMP is $∼O(\left(2^{N_{\Delta }+1}-1\right)N_{m}L_{x}+N_{\Delta }^{3})$. As the size of the grid for $\Delta R_{2}$ is $L_{\Delta }=2^{N_{\Delta }}$, $N_{\Delta }=\frac{\log L_{\Delta }}{\log2}$, and the operation count in terms of $L_{\Delta }$ is $∼O\left(L_{\Delta }N_{m}L_{x}+{\left(\frac{\log L_{\Delta }}{\log2}\right)}^{3}\right)$. We see that to leading order the computational cost is proportional to the size of the grid for $\Delta R_{2}$.

For the case of general *M* this cost is multiplied by (M−1).
